# Influence of Previous Infestation of Wheat Leaves and Ears by *Sitobion avenae* on Interaction with *Rhopalosiphum padi*

**DOI:** 10.3390/insects15110871

**Published:** 2024-11-06

**Authors:** Andreas Bühler, Rabea Schweiger

**Affiliations:** 1 Department of Chemical Ecology, Bielefeld University, D-33615 Bielefeld, Germany; andreas.buehler@uni-bielefeld.de; 2 Joint Institute for Individualisation in a Changing Environment (JICE), University of Münster and Bielefeld University, D-33615 Bielefeld, Germany

**Keywords:** interspecific herbivore–herbivore interactions, competition, facilitation, niche construction, aphids

## Abstract

Different herbivorous species that share a host plant may interact via competition or facilitation, depending on whether the interaction partners are hindered by or benefit from the interaction. Sap-sucking insects, such as aphids, can influence each other indirectly by altering the composition of the shared phloem sap. Aphid-induced changes in the plant may affect aphid performance and lead to a shift in the balance between different co-occurring aphid species. In this study, we compared the performance of the English grain aphid (*Sitobion avenae*) and the bird cherry-oat aphid (*Rhopalosiphum padi*) simultaneously infesting leaves or ears of wheat (*Triticum aestivum*) plants, which had been either previously infested by *S. avenae* or kept uninfested. Colonies of *S. avenae* were larger on ears than on leaves, while the opposite pattern was found for *R. padi*. Pre-infestation of ears, but not of leaves, by *S. avenae* led to a higher total aphid number and colony size of *S. avenae* at some time points. The balance between the two species was only slightly affected by previous infestation at some time points. The findings of this study contribute to the understanding of plant–aphid as well as aphid–aphid interactions in agricultural fields.

## Simple Summary

Interactions betweoen living organisms of different species may be negative or positive for the interaction partners, as these species may hinder or help each other. Plant sap-feeding insects, such as aphids, may indirectly interact with other species feeding on the same plant at the same time by causing changes to the shared host plant and potentially affecting the performance of and the balance between the species. We compared the colony sizes of two species of grain aphids, which were infesting leaves or ears of wheat at the same time. The wheat plants had either been previously infested by one of the two species or kept uninfested, to determine if a previous infestation affects interaction with the other species. Colony sizes of the pre-infesting aphid species were increased on the previously infested plants at some time points. There were only minor changes in the balance of the two species. With the findings derived from this study, we can better understand the interactions between two important cereal pest species. Our findings are important from an agricultural perspective, as wheat is an important crop plant species that may suffer from infestation by different aphid species.

## 1. Introduction

Phytophagous insects of different species that share a host plant may interact in various ways, with such interspecific interactions including competition and facilitation [1]. Competition frequently occurs when species depend on the same resources, where co-occurring species with similar traits may accentuate differences in their traits (e.g., in resource use), leading to niche differentiation that reduces competition and allows co-existence [2,3,4,5]. On the other hand, co-occurring herbivores may facilitate each other, with at least one species benefiting from the presence of the other, for example through easier resource access. Interspecific herbivore–herbivore interactions were found to be direct or indirect, with the shared host plant being an important mediator of indirect interactions, as different herbivores may affect each other by changing plant traits such as plant chemistry or morphology [6,7,8]. Such indirect, plant-mediated effects may also explain interactions between herbivores that are spatially and/or temporally separated. The outcome of herbivore-induced changes in plants for the herbivore community depends on the impacts of the changes on the different herbivore species.

Among phytophagous insects, interspecific competition has been shown to be especially strong between sap-feeding herbivores such as aphids [1,9]. Several studies on the performance of two aphid species simultaneously or subsequently infesting the same plant have been previously conducted. For example, in pecan aphids one species (*Monellia caryella*) negatively affected another species (*Melanocallis caryaefoliae*) subsequently infesting the same leaf, with no effects in the opposite direction [10]. *Myzus persicae* and *Aphis gossypii* attained similar colony sizes whether reared separately or simultaneously on the same plants, but with different within-plant distributions [11]. One species of pine aphids (*Eulachnus agilis*) showed higher growth rates and survival if shoots were infested by another pine aphid species (*Schizolachnus pineti*) [12]. Interactions between aphid species seem to be species-specific, with the timing and location of the infestation probably contributing to the final outcome of the interaction.

Aphid–aphid interactions may be due to direct effects between the aphids or to indirect effects, including aphid-induced modifications of the defenses (induction or suppression) and/or nutrients of their host plants that could also mediate interactions between spatially or temporally separated aphids. Such changes are most relevant if they occur in the phloem sap, as aphids rely on it for nutrition, especially regarding (essential) amino acids [13,14,15]. Aphids have been shown to reduce plant defenses via saliva-derived protein effectors [16,17] as well as to increase the concentrations and/or change the relative composition of (essential) amino acids [18,19]. It has been shown that the effects of a previous infestation by aphids on conspecifics and on heterospecific aphids range from positive to negative [10,20,21,22,23,24]. Studies with positive effects of previous infestation on conspecifics are evidence for niche construction, the process by which organisms modify their environment in ways enhancing their fitness [25,26,27,28]. However, heterospecific aphids may also benefit from the environmental modifications, for example from increased concentrations of (essential) amino acids after aphid infestation. Thus, next to direct interactions between aphids of different species, aphid-induced changes in the host plants (i.e., indirect effects) may lead to shifts in the abundance of heterospecific aphids. Indirect effects between aphids and chewing–biting herbivores have also been described [29,30]. More studies are needed to investigate the outcome of environmental modifications by an aphid species in the context of interspecific interactions like aphid–aphid interactions, as well as to assess under which circumstances these interactions are positive, neutral or negative for different species.

The aphid species *Rhopalosiphum padi* (bird cherry-oat aphid; Aphididae) and *Sitobion avenae* (English grain aphid; Aphididae) are economically important pests on cereals, including wheat [23,31]. While *S. avenae* prefers and better performs on ears of wheat [32,33,34], *R. padi* prefers and better performs on leaves and stems of this host species [35,36]. We have recently reported evidence for plant part-specific niche construction by *S. avenae* on wheat [37,38]. While on wheat leaves the performance of *S. avenae* was only slightly and transiently increased by a previous infestation with conspecifics [37], on wheat ears there was a strong and long-lasting positive effect of previous infestation, probably partly due to aphid-induced metabolic changes (e.g., higher relative concentration of asparagine, changes in relative concentrations of some specialized metabolites) observed in the phloem exudates of the ears [38]. The question remained whether there are also positive effects of previous infestation by conspecifics on *S. avenae* in the presence of heterospecific, wheat-infesting aphids such as *R. padi*. These aphids may be affected by the infestation by *S. avenae* as well and thus modify the response of *S. avenae* to a previous infestation by conspecifics. Since these two cereal aphids show a clear separation in their preferred niche on wheat [32,33,34,35,36], it is possible that each species shows a higher competitive ability against the other on its preferred plant part. Aphid-induced changes in their shared host plant that affect both species differently may shift the balance between the two species.

In this study, we aimed to investigate the effects of a previous infestation of wheat leaves or ears by *S. avenae* on conspecifics as well as on *R. padi* aphids simultaneously colonizing the same plant parts. Based on their preferred plant parts (see above), we expected higher colony sizes of *S. avenae* on ears than on leaves, with an opposite pattern for *R. padi*. Based on earlier studies [37,38], we predicted a small or no effect of previous infestation by *S. avenae* on colony sizes of this species on leaves, but higher colony sizes on pre-infested ears. Based on assumed similar nutritional requirements for *R. padi* as for *S. avenae*, we expected similar effects on *R. padi*. Finally, assuming that effects of niche construction are more beneficial to conspecifics than to heterospecifics, previous infestation by *S. avenae* should lead to a higher proportion of *S. avenae*, especially on ears.

## 2. Materials and Methods

The experiment, including plant cultivation and aphid rearing, was performed in a climate chamber at 20 °C, 70% relative humidity and 16:8 h light:dark.

### 2.1. Plant Cultivation

Seeds of spring wheat (*Triticum aestivum* L., cv. Tybalt, Poaceae; von Borries-Eckendorf, Leopoldshöhe, Germany) were germinated in a mixture of two parts river sand and one part soil (Fruhstorfer Spezialsubstrat Type P; Hawita Group, Vechta, Germany). The substrate had been steamed at 90–95 °C. After 7 days, individual seedlings were transferred to 2 L pots with the same substrate type. Plants were watered three times a week with tap water (equal amounts per pot), with amounts of water increasing over time along with the growth of the plants. Between 34 and 63 days after sowing, each plant was fertilized with 1.5 g mineral fertilizer (Plantosan N-P_2_O_5_-K_2_O 20-10-15, containing 6% MgO, 2% S and traces of B, Cu, Fe, Mn, Mo and Zn; Manna, Düsseldorf, Germany).

### 2.2. Aphid Rearing

*Sitobion avenae* Fabricius (Aphididae) aphids were purchased from re-natur (Stolpe, Germany), originally being from a conventional wheat field in Schleswig-Holstein (Germany), with the rearing supported as necessary with aphids from Katz Biotech (Baruth, Germany) that were from a laboratory population of the University of Göttingen. *Rhopalosiphum padi* L. (Aphididae) aphids were bought from Katz Biotech. The aphids were kept in gauze tents (50 × 50 × 50 cm) for 2–4 months (*S. avenae*) and for 1 month (*R. padi*) before monoclonal lineages were established for *S. avenae* (see below). All aphids were kept on 10–14-day-old wheat (cv. Tybalt) seedlings, which were refreshed weekly and cultivated under the same conditions as described above. Under these conditions, only clonal, apterous (wingless) females were produced. For *S. avenae*, monoclonal lineages were established by placing single adult females in gauze cages (diameter 20 cm, height 40 cm) containing pots with 15–20 2-week-old wheat (cv. Tybalt) seedlings. Ten lineages were produced and aphids of these lineages were allowed to freely reproduce for 2 months, with weekly refreshing the plants. Thus, aphid rearing plants were a maximum of 21 days old and did not flower. The cages were separated from each other by water barriers.

### 2.3. Infestation of Plants with S. avenae

**Figure 1:**
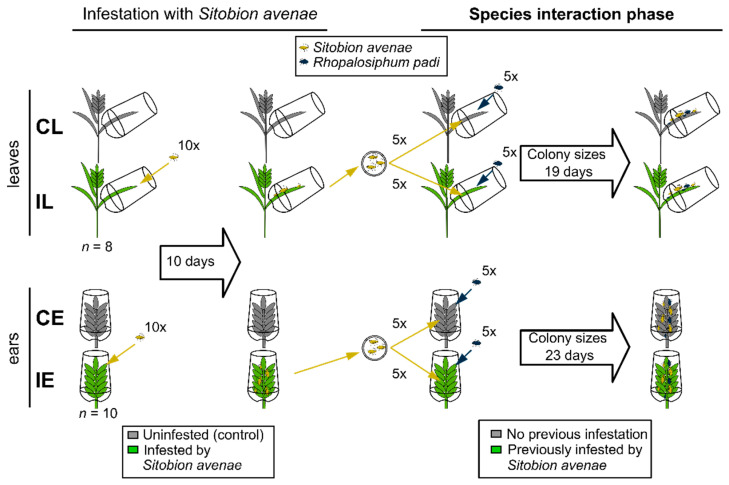
Experimental setup. In the first phase (**left**), *Triticum aestivum* plants were either left uninfested (control, gray) or infested with *Sitobion avenae* (green), on either the leaves (**top**) or the ears (**bottom**). After 10 days, *S. avenae* colonies were removed and nymphs were redistributed by placing five nymphs on the corresponding plant part in each of the cages of the different treatments. Simultaneously, five nymphs of *Rhopalosiphum padi* were added to all plants. During the species interaction phase (**right**), the colony sizes of the two aphid species were assessed over time. The abbreviations for the different treatment groups are given on the left.

Seventy-four days after sowing, when all plants had 1–3 flowering ears, plants were equipped with aphid cages made from PET cups with lids (WIMEX s.r.o, Náchod, Czech Republic; 500 mL volume, 95 mm diameter). The bottom of each cup was replaced with a fine metal mesh for air exchange, while the opening of the lid was covered with a piece of 2 mm white foam rubber, in which a slit had been cut through which the plant parts could be inserted. Plants were divided into four treatment groups: uninfested (control) leaf and *S. avenae*-infested leaf (CL and IL, *n* = 8) and uninfested (control) ear and *S. avenae*-infested ear (CE and IE, *n* = 10) and either the second-youngest leaf (i.e., the leaf directly under the flag leaf) of the main shoot (CL, IL) or the ear of the main shoot (CE, IE) of the wheat plant were inserted into the cage through the lid (Figure 1). For the infestation groups (IL, IE), cages were opened and 10 *S. avenae* nymphs (mid-instar) of the same monoclonal lineage were carefully put on the foam rubber of the lid next to the plant using a fine brush. Control (CL, CE) cages were treated similarly with a brush, but without adding aphids. On leaves, 8 clonal lineages were used (*n* = 8), while on ears all 10 lineages (*n* = 10) were used. The difference in sample sizes was due to space constraints, as the cages had to be attached horizontally to the leaves and vertically to the ears, i.e., leaf replicates (CL, IL) needed more space. Cages were closed, plants were placed in a randomized block design and the aphids were left in the cages for 10 days.

### 2.4. Species Interaction Phase

**Figure 2:**
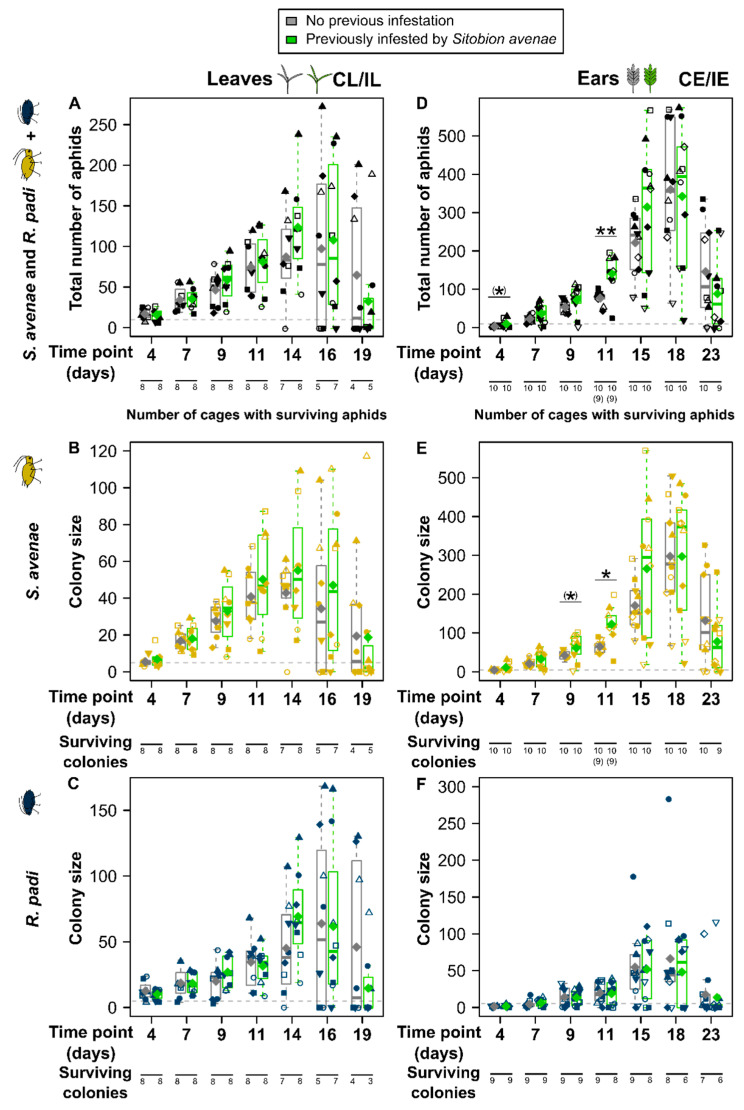
Numbers of aphids on *Triticum aestivum* leaves (**A**–**C**, left column) and ears (**D**–**F**, right column) for both *Sitobion avenae* and *Rhopalosiphum padi* (**A**,**D**; black), only *Sitobion avenae* (**B**,**E**; orange) and only *R. padi* (**C**,**F**; blue). Both aphid species simultaneously infested the plants in the species interaction phase. Data are given for different time points during the species interaction phase, with the initial numbers of aphids (i.e., five per species) being indicated with horizontal dashed lines. Plants either had no previous infestation (gray, treatment groups CL and CE; within time points, on the left) or had been previously infested by *S. avenae* (green, IL and IE; within time points, on the right). Data are shown as box-whisker plots with interquartile ranges (IQR; boxes) including medians (horizontal lines), means (large, filled diamonds) and whiskers (extending to the most extreme data points with maximum 1.5 times the IQR). Raw data points are shown as small, colored symbols; the same symbol type relates to the same clonal lineage of *S. avenae* and, within the same treatment group but at different time points or for the different species, it means that data points belong to the same aphid cage. The axes are differently scaled depending on the largest total aphid colony size. Within time points and plant parts, colony sizes of *S. avenae* were compared between previously infested and uninfested plants using paired *t*-tests or Wilcoxon signed-rank tests, while for total aphid numbers and for colony sizes of *R. padi* two-sample *t*-tests, Welch *t*-tests or Mann–Whitney *U*-tests were applied: (*) marginally significant at *p* < 0.1, * *p* < 0.05, ** *p* < 0.01, non-significant differences not indicated. For leaves *n* = 8, for ears *n* = 10. For the total number of aphids and the colony size of *S. avenae*, two data points (11 days, CE and IE) were omitted (see Materials and Methods), thus reducing the sample size from *n* = 10 to *n* = 9 here (indicated in parentheses).

After 10 days, all *S. avenae* aphids and their exuviae were carefully removed from the plants with a fine brush and collected, while control plants were brushed for the same amount of time. Fresh cages were attached to the plants. Then, mid-instar nymphs of the collected *S. avenae* aphids were redistributed, applying five nymphs to the corresponding plant part of each cage of the different treatment groups (Figure 1). Simultaneously, five mid-instar *R. padi* nymphs were taken from the rearing tent and were added to each cage. The species interaction phase was started with few aphids to avoid crowding and allow rapid colony growth; moreover, in cereal fields colonies usually start with few nymphs. The same number of aphids of both species was used to ensure similar starting conditions and avoid a competitive advantage of one species. All aphids were carefully put on the foam rubber next to the plants with a fine brush. The colony sizes of both aphid species were assessed by counting aphids, separately for both species, on the leaves after 4, 7, 9, 11, 14, 16 and 19 days and on the ears after 4, 7, 9, 11, 15, 18 and 23 days. For aphids, colony sizes of clonal groups are a valid measure of aphid fitness, as these herbivores reproduce parthenogenetically [39]. The experiments with leaves and ears were ceased at different time points, as leaves showed signs of senescence earlier and aphid colonies started to decline earlier on these plant parts. The experiments were terminated shortly after colony sizes began to decline (Figure 2).

### 2.5. Statistical Analyses

All analyses were performed in R 3.6.1 [40] using the packages *car* [41] and *rstatix* [42]. For all statistical tests, data were tested for normal distribution using Shapiro–Wilk tests and for homogeneity of variances using Levene tests. The colony sizes of *S. avenae* and of *R. padi*, the total number of aphids (i.e., the sum of both species) as well as the proportion of *S. avenae* (in relation to the total number of aphids, only for cages where surviving aphids of at least one species were present) were compared between plants that had been previously uninfested or infested by *S. avenae*. This was done separately for each time point and separately for leaves and ears, i.e., comparing the groups IL versus CL as well as IE versus CE within time points. For *S. avenae*, the colony sizes and proportions of aphids belonging to the same clonal lineage were compared between previously uninfested and infested plants using statistical tests for paired data (paired *t*-test or Wilcoxon signed-rank test). For other comparisons, i.e., total aphid numbers and colony sizes of *R. padi*, statistical tests for unpaired data (two-sample *t*-test, Welch *t*-test or Mann–Whitney *U*-test) were used. Thresholds of α = 0.05 for significance and α = 0.1 for marginal significance were applied for all tests. For one data point (*S. avenae*, 11 days, IE group) no aphids were found at that time point but at the next observation date 19 aphids were seen, indicating that a low number of aphids had been overlooked. Next to this data point, due to partly paired statistical tests (see above), the corresponding data point in the previously uninfested ears (*S. avenae*, 11 days, CE group) was removed from the data set (total number of aphids, colony size *S. avenae*, proportion *S. avenae*).

## 3. Results

We found large variation in aphid numbers (i.e., the sum of *S. avenae* and *R. padi* aphids) as well as the colony sizes of the two aphid species, even within plant parts and treatments, especially at later time points where colony sizes ranged from 0 to more than 100 (leaves, both species) and more than 500 (ears, *S. avenae*) (Figure 2).

**Figure 3:**
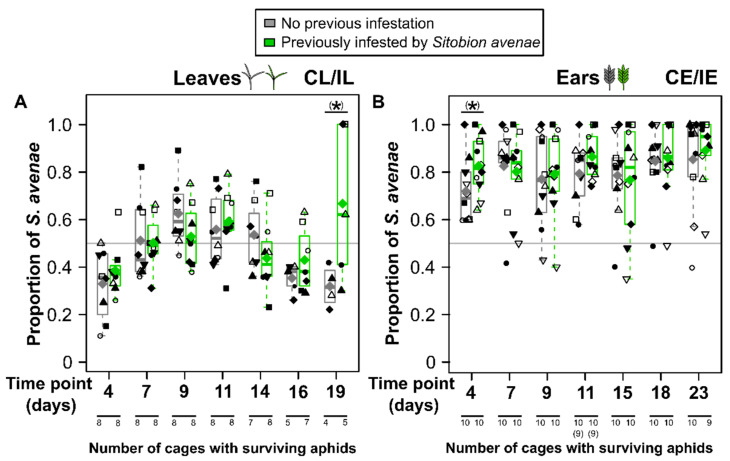
Proportion of *Sitobion avenae* on *Triticum aestivum* leaves (**A**) and ears (**B**), based on the total number of aphids of the two simultaneously infesting species *S. avenae* and *Rhopalosiphum padi*. Data are given for different time points during the species interaction phase. Plants either had no previous infestation (gray, treatment groups CL and CE; within time points, on the left) or had been previously infested by *S. avenae* (green, IL and IE; within time points, on the right). Data are shown as box-whisker plots with interquartile ranges (IQR; boxes) including medians (horizontal lines), means (large, filled diamonds) and whiskers (extending to the most extreme data points with maximum 1.5 times the IQR). Raw data points are shown as small, colored symbols; the same symbol type relates to the same clonal lineage of *S. avenae* and, within the same treatment group but at different time points or for the different species, it means that data points belong to the same aphid cage. The horizontal gray lines indicate a proportion of 0.5, i.e., equal colony sizes of the aphid species. Within time points and plant parts, proportions were compared between colonies on previously infested and uninfested plants using two-sample *t*-tests or Mann–Whitney *U*-tests: (*) marginally significant at *p* < 0.1, non-significant differences not indicated. As cages without surviving aphids of at least one species were excluded, the original sample sizes (for leaves *n* = 8, for ears *n* = 10) were reduced as indicated at the bottom. Two data points (11 days, CE and IE) were omitted (see Materials and Methods), thus reducing the sample size from *n* = 10 to *n* = 9 here (indicated in parentheses).

On leaves, in most cages the total aphid numbers increased until 14 days after beginning of the species interaction phase, reaching values of up to 272 aphids per cage (Figure 2A). During this time, leaves became increasingly yellow and some began to dry out. At day 16, no further increase of aphid numbers was seen, while at day 19 aphid numbers had decreased and only 4 out of 8 control leaves and 5 out of 8 previously *S. avenae*-infested leaves had any surviving aphids left. A similar pattern over time was found for both aphid species (Figure 2B,C). While for *S. avenae* a maximum colony size of 117 per leaf cage was found, *R. padi* showed a maximum of 168 aphids per leaf cage. Neither the total aphid numbers nor the colony sizes of any of the two aphid species significantly differed between leaves previously infested by *S. avenae* and previously uninfested control leaves at any time point. However, for the total number of aphids as well as for *S. avenae* colony sizes, means and medians were consistently slightly higher on previously infested plants on days 7 to 16, while at day 19 it was the other way round. A similar tendency was seen for *R. padi*, but only for some time points. The colony sizes of the two aphid species on the leaves were similar, as indicated by a proportion of *S. avenae* close to 0.5, if averaged over time (Figure 3A). On day 4, *S. avenae* aphids were on average slightly outnumbered by *R. padi*, with the ratio later shifting to slightly more *S. avenae* (days 9, 11) and back to more *R. padi* (day 16) aphids. There were no significant differences in the proportions of *S. avenae* between previously *S. avenae*-infested and uninfested leaves, except for day 19, where the proportion of *S. avenae* was slightly (marginally significant) higher if the leaves had been previously infested by *S. avenae* (two-sample *t*-test, *t*_7_ = 2.06, *p* = 0.079). At this time point, in two cages *R. padi* but not *S. avenae* colonies had died out (i.e., proportion of *S. avenae* of 1).

The ears showed considerably higher aphid numbers than leaves, with up to 576 aphids per cage (Figure 2A,D). The ears began to turn yellow and grains began to ripen towards the end of the species interaction phase. The colony sizes of *S. avenae* were much higher on ears than on leaves (Figure 2B,E), reaching up to 569 aphids. For *R. padi*, differences between ears and leaves were less obvious, but on average colony sizes were higher on the leaves (Figure 2C,F). The total aphid numbers as well as the colony sizes of the two aphid species increased until 18 days after the beginning of the species interaction phase, with a drop in aphid numbers at day 23. Most colonies were still alive at the end of the experiment. There was only a single, previously *S. avenae*-infested ear on which all aphids had died out at day 23. For *R. padi*, one colony of each treatment group had already died out at 4 days, while at day 23 three (control ears) and four (previously *S. avenae*-infested ears) colonies had died out. Mean and median numbers of total aphids and of *S. avenae* were (slightly) higher on ears previously infested by *S. avenae* until day 15, with the trend reversing by day 23 (Figure 2 D,E). These differences were (marginally) significant on day 4 (total aphid number: Mann–Whitney *U*-test; *W = 26*, *p* = 0.073), on day 9 (*S. avenae*: paired *t*-test, *t*_9_ = 1.88, *p* = 0.093) and on day 11 (total aphid number: two-sample *t*-test, *t*_16_ = 3.53, *p* = 0.003; *S. avenae*: paired *t*-test, *t*_8_ = 3.17, *p* = 0.013). For *R. padi*, there were no obvious trends or significant differences between the treatment groups (Figure 2F). The dominance of *S. avenae* over *R. padi* on the ears was reflected in proportions of *S. avenae* up to 0.99 in cages where *R. padi* did not die out entirely, with all medians and means being clearly larger than 0.5 (Figure 3B). At day 4 of the species interaction phase, there was a slightly (marginally significant) higher proportion of *S. avenae* on ears previously infested by conspecifics than on previously uninfested ears (two-sample *t*-test, *t*_18_ = 1.86, *p* = 0.080).

Though not quantified, there appeared to be some spatial separation of the aphids within the plant parts, with *R. padi* more likely colonizing the abaxial side of the leaves and the stalks of the ears, while *S. avenae* did not seem to show a preference for the abaxial or adaxial side of the leaves and on the ears was more likely found between the spikelets.

## 4. Discussion

We investigated how a previous infestation of wheat leaves and ears by *S. avenae* influences the performance of both conspecifics as well as of heterospecific *R. padi* aphids simultaneously colonizing the same plant parts.

The rise of aphid numbers over time both on leaves and ears indicates that phloem sap-located resources were likely abundant and readily accessible to the aphids early in the experiment. However, the following decrease of aphid numbers at the end of the experiment suggests that later a scarcity of resources may have led to increased intra- and interspecific competition, likely exacerbated by a loss of water during beginning senescence of the plant parts and grain ripening in the ears. Indeed, another study suggested that interspecific, symmetric competition between *S. avenae* and *R. padi* takes place on wheat [23]. Specifically, competition for feeding sites and phloem sap-located resources may play a role in this context. The decline of colony sizes may also have been due to the strong infestation by the two aphid species, which may have led to an increase in plant defenses. Under natural conditions, if the carrying capacity of a plant (part) is reached at high aphid densities and plant quality declines, aphids often migrate to other feeding sites, mainly as alatae (winged) morphs [43,44]. In our experiment only a few alatae were observed, but because winged morphs are only seen in the next generation, we cannot rule out that the induction of alatae had already happened.

As expected, colonies of *S. avenae* grew considerably larger on ears than on leaves, while *R. padi* colonies were bigger on leaves than on ears. This is in line with reports on the preference and performance of the two aphid species for/on these plant parts [32,33,34,35,36]. Because in our study the aphids of both species were restricted to one plant part and could not choose between plant parts, these results reflect performance differences between leaves and ears in an interspecific interaction scenario and are probably related to species-specific adaptations to the different host plant parts. In addition, in cereal fields there may be temporal and/or spatial niche differentiation between *S. avenae* and *R. padi*, as suggested for different aphid species [45,46]. The finding that *S. avenae* was the dominant species on the ears, while on the leaves *R. padi* did not show larger colony sizes than *S. avenae*, may be explained by the late growth stage of the wheat plants. Indeed, in spring *R. padi* aphids usually arrive earlier on cereal fields than *S. avenae* [47]. Thus, older wheat plants may be suboptimal hosts for *R. padi*, while they may still be good hosts for *S. avenae*. In general, the suitability of their host plants across the season probably largely contributes to the seasonal abundance patterns of cereal aphids observed in cereal fields [32,48]. In natural settings, the aphids could also choose between leaves and ears and also change between plant individuals. However, even without having these options, in our study there were indications for niche differentiation between the species within plant parts, especially in the ears, where *R. padi* mainly infested the stalks while *S. avenae* was mainly found between the spikelets. Indeed, niche differentiation between different plant parts and microhabitats within plant parts is known from other aphid species. For example, two aphid species that are monophagous on *Tanacetum vulgare*, i.e., *Macrosiphoniella tanacetaria* and *Uroleucon tanaceti*, showed performance optima on different plant parts, probably allowing niche differentiation [49]. *Aphis fabae* preferred leaves over stems as well as the abaxial over the adaxial side of leaves when *Acyrtosiphon pisum* was simultaneously infesting broad beans, while the distribution was different when raised separately [50]. Specifically, for cereal aphids, species-specific distribution patterns within wheat plants have been reported [46,51]. Niche differentiation is probably dependent on the plant traits (e.g., phloem sap chemistry) at the different feeding sites as well as on aphid species-specific adaptations to these.

In accordance with our expectations, there were signs of niche construction by *S. avenae* on wheat ears, but not/less on leaves, i.e., colonies on pre-infested wheat ears (but not/less on leaves) were larger at least at certain time points. This is in line with earlier studies reporting plant part-specific niche construction by *S. avenae* on wheat [37,38]. The underlying mechanisms may be, for example, suppressed plant defenses, increased amino acid concentrations and/or a different amino acid composition with a higher percentage of essential amino acids after aphid infestation. In the previous study on wheat ears, pre-infestation by conspecifics led to strong and long-lasting positive effects on *S. avenae* colony sizes [38], while in the current study the positive effects were only slight and transient. This may indicate that the co-occurring *R. padi* aphids attenuated the beneficial impact of *S. avenae* on conspecifics. Based on assumed similar nutritional requirements, we also expected positive effects of a previous infestation by *S. avenae* on heterospecific *R. padi* aphids, although to a lesser extent compared to the impacts on conspecific *S. avenae*, resulting in a higher proportion of *S. avenae*. However, there were no significant differences in *R. padi* colony sizes between the infestation treatments. This indicates that in an interspecific interaction scenario with *S. avenae*, *R. padi* does not benefit from previous infestation by *S. avenae*; however, it is possible that, as for *S. avenae*, there would be beneficial effects of previous infestation by *S. avenae* on *R. padi* if the species were tested separately. In other studies, both positive and negative effects, as well as no effects, of aphid infestation on subsequently colonizing heterospecific aphids have been found [10,21,23,52]. For the aphid species investigated in our study, negative effects of previous infestation by *R. padi* on the reproductive rate of *S. avenae* have been reported on wheat seedlings and tillering plants, while on flag leaves a previous infestation by *S. avenae* did not affect *R. padi* [23]. For the case that *R. padi* and *S. avenae* co-occurred from the beginning on wheat seedlings or tillering plants (i.e., an experimental design differing from the one applied in the current study), the reproductive rate of either species was decreased in the presence of the other one, with *R. padi* showing higher reproduction than *S. avenae* [23]. Differences between the studies may be due to the fact that in our study, in contrast to the study by Gianoli [23], both aphid species were restricted to the same plant part. Whether there are plant-mediated effects of aphids on con- and heterospecifics probably largely depends on the aphid-induced changes in the host plant as well as on the nutritional requirements of the aphid species. It is known that plant responses to aphids depend on the aphid species [19,53,54] and that aphid species differ in their survival on different diets [55] and in their amino acid budgets [56]. In contrast to our expectation, the proportions of *S. avenae* were only slightly higher on pre-infested plant parts at only one time point each (leaves: day 19; ears: day 4). For the interaction of *S. avenae* and *R. padi* on wheat, the nutritional requirements of these two aphid species as well as their competitive strengths are important. The slightly higher proportion of *S. avenae* on pre-infested leaves at the end of the experiment when total aphid numbers were already declining was partly due to *S. avenae* colonies outliving extinct *R. padi* colonies. This may indicate that niche construction in an interspecific interaction context only becomes noticeable during competition for scarce resources, with previous niche construction by conspecifics helping *S. avenae* to endure for longer than *R. padi*. Moreover, as *R. padi* usually arrives earlier in cereal fields than *S. avenae* [47], it may be more common for *S. avenae* to invade leaves currently or previously infested by *R. padi* than vice versa and thus *S. avenae* may be better adapted to outcompete *R. padi* to establish colonies on already infested plants. Better survival on senescing leaves may represent a fitness advantage for *S. avenae* due to their late arrival in the season, when they use the senescing leaves only as a temporary niche until ears, which they prefer and on which they perform better [32,33,34], emerge. In contrast, the earlier-arriving *R. padi* is probably more adapted to younger leaves. Interspecific effects may also vary with colony size; for example, infestation of wheat by small colonies of *S. avenae* increased the fecundity of *R. padi*, but larger colonies had the opposite effect [57]. To account for the preferred plant parts and for the order of arrival in cereal fields, future studies should investigate niche construction by *R. padi*, assessing effects of previous infestation by this species on conspecifics as well as on interacting heterospecific *S. avenae*. Such studies should also take into account the preferred niches of the two aphid species, by testing whether previous and simultaneous infestation of the lower leaves and stem of wheat by *R. padi* affect *S. avenae* infesting the ears.

Aphid–plant and interspecific aphid–aphid interactions are highly complex and variable at many levels, making it difficult to generalize any interactions. The metabolic composition of phloem sap/exudates of plants differs between plant species, plant parts as well as chemotypes and depends on plant age [14,49,58]. The interaction of aphids with plants highly depends on chemical traits of the plants, with amino acids playing an important role [14,56,59]. However, other compound classes are relevant as well; for example, differences in flavonoids between wheat cultivars may affect the performance of *S. avenae* [60]. Within aphid species, genotypes can be highly variable, showing distinct nutrient requirements, performances, preferences as well as salivary effectors [61,62,63,64,65]. This variability in plants and aphids means that aphid–plant interactions are often highly dependent on specific conditions. For example, the induction of certain metabolites in *Medicago truncatula* depends on the genotypes of both the aphid and the plant, on aphid density as well as on the duration of infestation [66]. Factors related to climate change may also influence aphid–plant and aphid–aphid interactions. For example, the dominance hierarchy between *S. avenae* and *R. padi* has been shown to be influenced by warming and elevated CO_2_, in favor of *R. padi* [67,68]. A temperature increase was also found to change the competitive interaction between these two aphid species and to decrease the niche breadth of *S. avenae* when interacting with *R. padi* [69]. More studies with different temperature regimes are needed to better understand the tripartite interaction (wheat, *S. avenae*, *R. padi*) under climate change. Such studies should also include the assessment of impacts of temperature on the development of wheat, for example the emergence of ears during the season, which is crucial for *S. avenae*. Natural enemies may further shape the interaction between the two aphid species. In agricultural wheat fields, the wheat cultivar as well as the management techniques probably also influence the success of these aphid species. Thus, it is possible that niche construction effects on con- and/or heterospecific aphids in an interspecific interaction appear under different conditions than tested in the current experiment. In addition to the factors listed above, our previous studies [37,38] and the current study emphasize that the effects of previous infestation by conspecific aphids may depend on the presence of other aphid species, contributing to the understanding of the complex ecological plant–aphid–aphid interactions in cereal fields. Future studies on niche construction by aphids should be based on larger samples sizes, as some effects may have been masked by the huge variability in aphid numbers and colony sizes in the current study. Taken together, our study contributes to the understanding of plant–aphid–aphid interactions in an agricultural context and may be used for the improvement of pest monitoring and management.

## Data Availability

Data are contained within the article.
